# ROS accelerates the progression of hypertrophic cardiomyopathy

**DOI:** 10.1016/j.gendis.2025.101741

**Published:** 2025-06-27

**Authors:** Jinhua Cao, Yafei Zhai, Ke Li, Jiajv Li, Xiaoxu Tian, Jianchao Zhang, Shuang Li, Mengduan Liu, Xiaowei Li, Jianzeng Dong, Xiaofang Wang

**Affiliations:** a Department of Cardiology, The First Affiliated Hospital of Zhengzhou University, Zhengzhou, Henan 450052, China; b Henan Key Laboratory of Hereditary Cardiovascular Diseases, Zhengzhou, Henan 450052, China

**Keywords:** Hypertrophic cardiomyopathy, Induced pluripotent stem cell, Melatonin, MYBPC3, Oxidative stress

## Abstract

MYBPC3 mutations are the leading cause of hypertrophic cardiomyopathy. Here, to study the pathogenesis of hypertrophic cardiomyopathy, we created a MYBPC3 knockout (KO) model using human induced pluripotent stem cell-derived cardiomyocytes (hiPSC-CMs). MYBPC3-deleted hiPSC-CMs revealed the characteristics of heart failure, which exhibited increased contractility at 30 days but decreased at 40 days. Furthermore, at 40 days, it also shows abnormal calcium handling, increased ROS levels, and mitochondrial damage. Further RNA sequencing revealed that the oxidative stress pathway was aberrant, in addition to alterations linked to hypertrophic cardiomyopathy. Moreover, after adding melatonin to hiPSC-CMs at 30 days, MYBPC3-deleted hiPSC-CMs showed restored calcium handling capacity, decreased ROS levels, and improved myocardial contractility. In summary, reducing ROS can improve the phenotype of hypertrophic cardiomyopathy.

## Introduction

Hypertrophic cardiomyopathy (HCM), an inherited genetic disease, occurs in 0.2%–0.5% of cases.^1^ HCM is a disease characterized by diastolic dysfunction, fibrosis, and asymmetric thickening of the left ventricle.^2^ HCM is considered the top reason for sudden cardiac death among young athletes without preceding symptoms and is also a leading cause of heart failure.^3^ So far, there is no effective treatment to slow the progression of the disease, although mavacamten has shown promise in reducing left ventricular hypertrophy.^4^^,^^5^ Adverse clinical outcomes of HCM increase with the duration of the disease, thus emphasizing the importance of therapeutic strategies to slow disease progression.^6^

**Figure 1:**
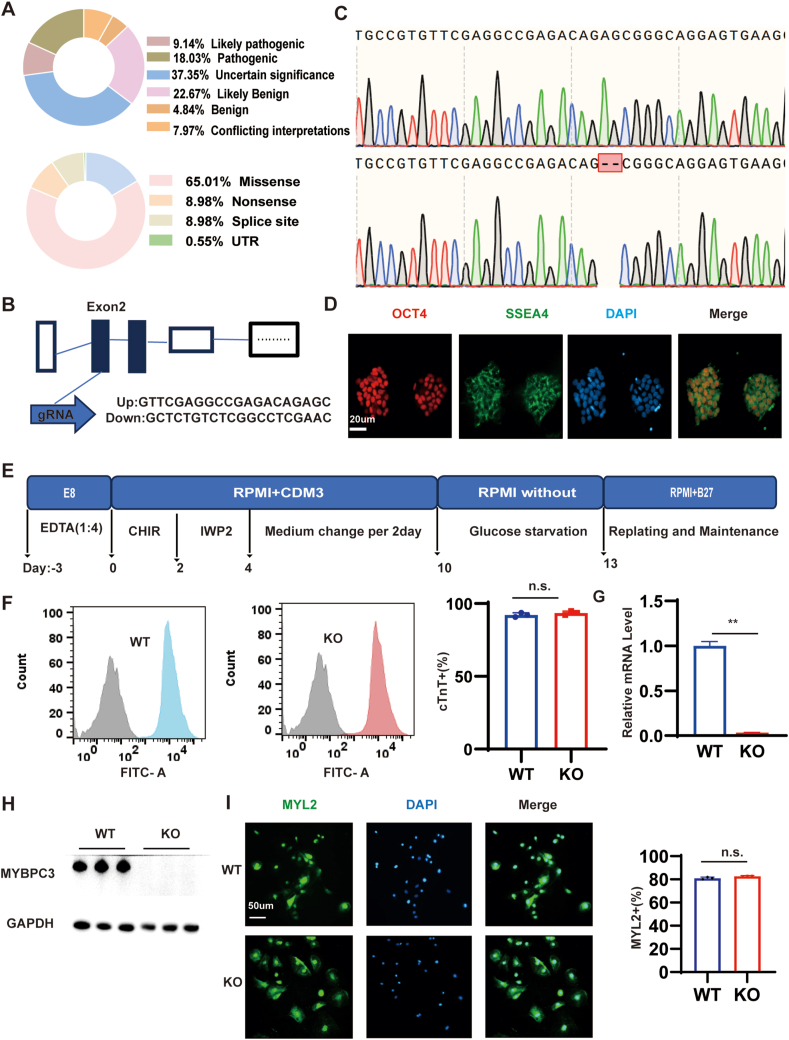
Generation of MYBPC3-KO hiPSCs. **(A)** The incidence data of MYBPC3 in hypertrophic cardiomyopathy were summarized, and the results were obtained from the ClinVar database. **(B)** The structure of the MYBPC3 gene and the location of gRNA for epiCRISPR/Cas9 editing. **(C)** Sequencing chromatograms verified a homozygous MYBPC3-KO hiPSC line, characterized by a deletion of 2 nucleotides in each allele. **(D)** Immunostaining of MYBPC3-KO colonies for the pluripotency markers OCT4 and SSEA4. Scale bar, 20 μm. **(E)** Molecule-based methods were used to induce cardiac differentiation. **(F)** Analysis for cTnT from WT and MYBPC3-KO differentiation protocols before purification at day 10. **(G, H)** Quantitative real-time PCR and western blotting analysis of MYBPC3 gene in both WT and MYBPC3-KO hiPSC-CMs at day 20. **(I)** Immunostaining for expression of MYL2 in WT and MYBPC3 KO CMs at day 40. Scale bar, 50 μm. The results were presented as mean ± standard deviation of three independent experiments. ∗*p* < 0.05; ∗∗*p* < 0.01; n.s. means no significance. MYBPC3, myosin binding protein C3; KO, knockout; WT, wild type; hiPSC, human induced-pluripotent stem cell; CM, cardiomyocyte; SSEA4, stage-specific embryonic antigen 4; OCT4, octamer-binding protein 4; cTnT, cardiac troponin T; MYL2, myosin light chain 2.

Cardiac myosin binding protein-C (cMyBP-C) is a vital part of the thick filaments found in almost all vertebrate striated muscles. Myosin binding protein C3 (MYBPC3) is the most frequently altered sarcomeric gene among HCM patients, and accounts for approximately 40%–50% of HCM; frameshift or nonsense mutations account for the highest proportion of MYBPC3 (Fig. 1A).^7^, ^8^, ^9^ In recent years, MYBPC3 mutation-caused HCM has been investigated, and *cMyBP-C*^*+/−*^ mice show asymmetric ventricular septal hypertrophy at about 10 months, but do not have the left ventricle dysfunction that is seen in human HCM. There were also studies showing that *cMyBP-C*^*+/−*^ mice were identical to *cMyBP-C*^*+/+*^ mice, but *cMyBP-C*^*−/−*^ mice showed pronounced heart hypertrophy at 3–5 months of age with significant systolic and diastolic dysfunction.^10^^,^^11^ In models using human induced-pluripotent stem cell-derived cardiomyocytes (hiPSC-CMs), *cMyBP-C*^*−/−*^ hiPSC-CMs present with contractile dysregulation, whereas *cMyBP-C*^*+/−*^ hiPSC-CMs exhibit proper contractile force associated with compensated MyBP-C levels.^12^ Most MYBPC3 mutations related to HCM are heterozygous in patients, often leading to late-onset disease; the course of the disease is usually benign.^13^ Numerous studies have demonstrated that only patients with two or more mutations always progress to a severe phenotype, similar to that observed in animal models. Types of mutations include heterozygous, double heterozygous, or homozygous.^10^^,^^14^, ^15^, ^16^

The mismatch between reactive oxygen species (ROS) production and the antioxidant system's ability is known as oxidative stress. In physiological situations, small amounts of ROS are crucial in signaling pathways and are cleared by the antioxidant system. Under pathological conditions, the antioxidant system is unable to remove excess ROS, and this excess can lead to DNA damage, and peroxidation of proteins and lipids, ultimately resulting in irreversible cell death, which is also common in diseases of the cardiovascular system.^17^ There are many causes of myocardial hypertrophy, including stress overload and genetic mutations. In terms of molecular mechanisms, oxidative stress has been identified as being involved in a variety of pathological myocardial hypertrophy processes, and ROS has been proven to make an important difference in the pathogenic mechanism of HCM.^18^, ^19^, ^20^, ^21^

Compensated myocardial hypertrophy is thought to be an adaptive process in which oxidative stress and antioxidant systems balance each other. However, when antioxidant activity is saturated with excess oxygen radicals or antioxidant levels are reduced, oxidative stress occurs, leading to heart dysfunction and heart failure. Oxidative stress is important during compensatory myocardial hypertrophy and heart failure. When excess ROS is generated, it can lead to heart dysfunction and heart failure.^22^, ^23^, ^24^, ^25^ Therefore, it is hypothesized that oxidative stress may relate to the course of HCM to heart failure. To verify whether it makes sense, we generated a model using hiPSC-CMs from a healthy volunteer.

## Materials & methods

### Reprogramming of hiPSCs and cell culture

Mononuclear cells from peripheral blood (PBMCs) were extracted following the method described earlier.^26^ Using the Sendai virus vector, these cells showed an increased expression of the four Yamanaka factors. Single cloning was chosen following three weeks of transfection. hiPSC cells were cultured in ncTarget (Nuwacell, China) complete medium on plates coated with 150 μg/cm^2^ Matrigel (ABW, China), undergoing daily changes of the medium in a moisture-rich 5% CO_2_ environment at 37 °C. For cellular transfer, 0.5 mM of ethylenediaminetetraacetic acid (EDTA; AM9261, Invitrogen, USA) was introduced once the cell density was attained between 75% and 80%. The Ethics Review Committee of Zhengzhou University's First Affiliated Hospital (2018-KY-38) approved this research.

### *In vitro* CM differentiation and purification

As previously described, hiPSCs are differentiated into CMs using a molecular-based approach defined by chemistry.^27^ Around 8–10 days after differentiation, spontaneous contractions of CMs can be observed, followed by purification using metabolic methods. For cardiomyocyte maintenance, the RPMI 1640 and B27 (60703ES10YESEN, China) medium were changed every 3 days.

### Editing of genomes

The sgRNA targeting exon 2 of MYBPC3 was designed with online tools (https://zlab.bio/guide-design-resources). sgRNA (GTTCGAGGCCGAGACAGAGC) was linked with the epi-CRISPR plasmid. 1 × 10^6^ hiPSCs were dissociated with 0.5 mmol/L EDTA, and a 3 μg plasmid was introduced into cells through electroporation. Subsequently, the cells were planted in 6-well plates layered with Matrigel and grown with the ncTarget containing 10 μM Y-27632. Transfected cells were screened with 0.2–1 μg/μL puromycin on day 3. After drug screening, the surviving colonies were planted in a 48-well plate to undergo additional validation through gene sequencing (Sangon Biotech, Shanghai, China). To produce hiPSC-GCaMP and MYBPC3-KO-GcaMP, the process involved electroporating 1 μg AAVS1-sgRNA plasmid (Addgene, #100554, USA) and 1 μg pAAVS1-PC-GCaMP6f plasmid (Addgene, #73503) into hiPSC and MYBPC3-KO hiPSC. The procedures for screening, selecting, and identifying puromycin colonies followed the previously outlined methods.

### Flow cytometry

Separating hiPSC-CMs were fixed with fixation buffer for 8 min, and then incubated with the anti-cardiac troponin T (anti-cTnT) in blocking solution for 1 h. Subsequently, the cells were washed three times and treated with Alexa Fluor 488 goat anti-mouse IgG at 37 °C for 1 h. The cells were quantified using a BD Accuri C6 plus flow cytometer (BD Biosciences, USA).

To measure mitochondria and ROS, separated hiPSC-CMs were stained with 50 nM of MitoTracker Green (40742 ES; YESEN, China), 100 μM CellROX Green (50101ES01; YESEN, China), and 0.5 mM of MitoSOX Red (40741ES50; YESEN, China). The live cells underwent incubation using the above dyes (made in RPMI 1640; HyClone) for 30 min in a cell culture incubator. The samples were tested using a BD Accuri C6 plus flow cytometer (BD Biosciences, USA) and then analyzed with the FlowJo V10 program.

### Immunostaining analyses

Cells were fixed with 4% paraformaldehyde and permeabilized using 0.2% Triton X-100 (Sigma, USA) for 8 min, and then blocked with 4% bovine serum albumin (Sigma) for 30 min. Subsequently, the cells underwent an overnight incubation with the primary antibody at a temperature of 4 °C, followed by incubation with a secondary antibody and DAPI (4′,6-diamidino-2-phenylindole) (Invitrogen, USA). Images were captured with a fluorescence microscope (ZEISS). Cell area was measured by ImageJ Pro. The antibodies are listed in Table S1.

### Quantitative real-time PCR

Cell cDNA was extracted as mentioned earlier.^26^ The quantitative real-time PCR process was conducted using Quant Studio 3 (Thermo) with TB Green Premix Ex Taq II (Takara). The comparative measurement of the target genes was determined using the 2^−ΔΔCt^ method, with glyceraldehyde-3-phosphate dehydrogenase (GAPDH) serving as the reference gene. Primer sequences are listed in Table S1.

### Western blotting

Cell pellets were lysed with RIPA Lysis Buffer (G2002, Servicebio, China), containing a protease inhibitor mixture (G2008, Servicebio, China) and phosphatase inhibitor mixture (G2007, Servicebio, China). The samples were resolved by SDS-PAGE and then transferred to the polyvinylidene fluoride membrane at 300 mA for 90–120 min. The membrane was blocked with 5% skim milk blocking solution at 37 °C for 1 h, followed by incubation with primary and secondary antibodies as detailed in Table S1. ImageJ was used to analyze and measure the intensity of the band signals.

### Calcium imaging

hiPSC-GCaMP were seeded onto a 12-well plate. Calcium transients were captured using an EMCCD camera with a fluorescence microscope and Hcell software.^28^ These results were analyzed with ImageJ. Time to peak, 50% decay time (50% time from peak to baseline), and amplitude were calculated.

### Myocardial contractility

Cells were cultured in a 6-well plate. Carl Zeiss was employed to record the videos. The pulsation of myocardial cells was captured and recorded for 3–5 s, preserved in the initial.czi file type, and subsequently transformed into an uncompressed.avi file type (70 frames per second). ImageJ was equipped with a uniqueins (MUSCLEMOTION) for analyzing videos,^29^ with the outcomes being processed and preserved.

### Statistical analyses

GraphPad Prism 9.0 and SPSS (version 25.0) were used to analyze data. Data were displayed as mean ± standard deviation. An unpaired two-tailed student's *t*-test was used when the two sets of data conformed to the homogeneity of variance. Otherwise, the Mann–Whitney *U* test was used. Significant differences were considered when *p*-values <0.05 (∗*p* < 0.05, ∗∗*p* < 0.01, and ∗∗∗*p* < 0.001).

## Results

### Generation of homozygous MYBPC3 KO hiPSCs and their differentiation into CMs

To create MYBPC3-KO hiPSC line using the CRISPR-Cas9 system, we designed a single-guide RNA targeting exon 2 of MYBPC3; and then the epi-CRISPR linking the gRNA was electroporated into peripheral blood-derived hiPSCs (Fig. 1B).^30^ After puromycin screening, the DNA of the colonies was expanded for PCR screening validation, obtaining a MYBPC3-KO clone with a two-base (GA) deletion, resulting in frameshift coding sequences, and obtaining homozygous MYBPC3-KO hiPSCs (Fig. 1C). We then confirmed that MYBPC3-KO hiPSCs manifested the pluripotency markers stage-specific embryonic antigen 4 (SSEA4) and octamer-binding protein 4 (OCT4) (Fig. 1D). We differentiated MYBPC3-KO hiPSC lines and the wild-type (WT) hiPSCs toward CMs (hiPSC-CMs) using a special protocol (Fig. 1E). After 20 days of differentiation, testing MYBPC3 by quantitative real-time PCR and western blotting, we found that the expression of MYBPC3 gene was significantly reduced and MYBPC3 protein was not expressed compared with the WT hiPSC-CMs (Fig. 1G, H). The impact of MYBPC3 deficiency on differentiation efficiency was then evaluated using flow cytometry. The MYBPC3-KO hiPSC-CMs were positive for the myocardial-specific marker cTnT and the ventricular marker myosin light chain 2 (MYL2); there was no statistical difference between MYBPC3-KO hiPSC-CMs and WT hiPSC-CMs (Fig. 1I). These results suggest that hiPSC-CMs with MYBPC3 deficiency can differentiate into CMs similarly to the WT hiPSCs.

### MYBPC3-KO hiPSC-CMs show abnormal cardiac calcium handling and myocardial contractility

**Figure 2:**
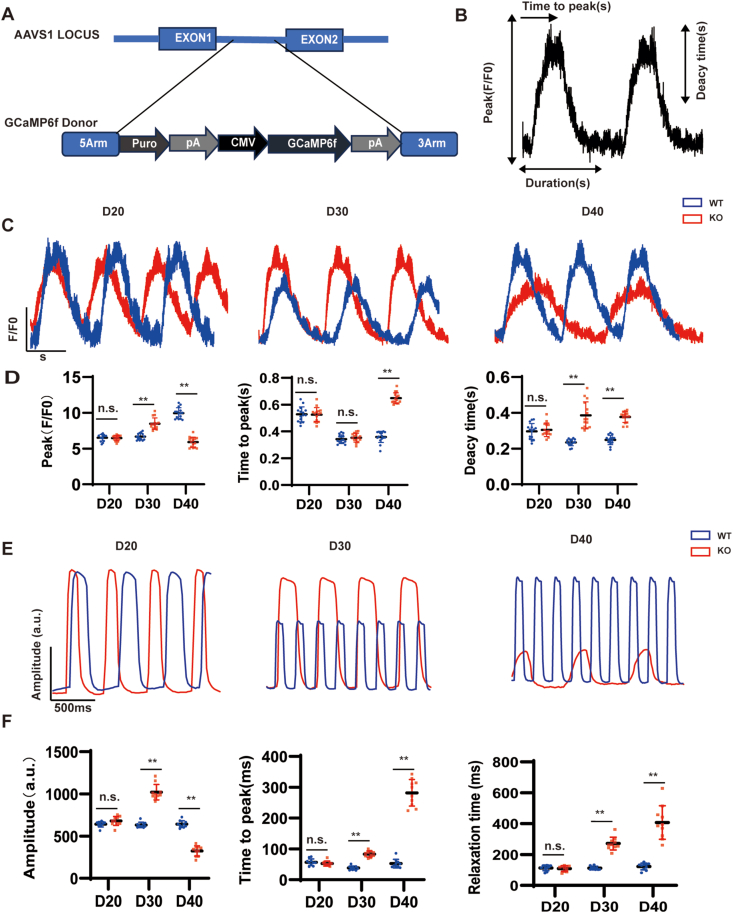
MYBPC3-KO hiPSC-CMs show abnormal cardiac calcium handling and myocardial contractility. **(A)** The diagram illustrates the integration of the GCaMP-expression cassette into the AAVS1 site of both WT and MYBPC3-KO hiPSC-CMs through nickase CRISPR/Cas9 editing. **(B)** Calcium transients averaged over space, displaying parameters observed for assessing calcium handling. **(C)** Illustrative line-scan visualizations for WT-GCaMP and MYBPC3-KO-GCaMP hiPSC-CMs at day 20, 30, and 40. **(D)** Quantification of peak, time to peak, and calcium transient duration in WT and MYBPC3 KO hiPSC-CMs (*n* = 12 cells per group). **(E)** Images of myocardial contractility in WT and MYBPC3-KO hiPSC-CMs at day 20, 30, and 40. **(F)** Quantification of amplitude, time to peak, and relaxation time in WT hiPSC-CMs and MYBPC3-KO hiPSC-CMs (*n* = 12 cells per group). ∗*p* < 0.05; ∗∗*p* < 0.01; n.s. means no significance. MYBPC3, myosin binding protein C3; KO, knockout; hiPSC, human induced-pluripotent stem cell; CM, cardiomyocyte; GCaMP, green fluorescent calcium-modulated protein; AAVS1, adeno-associated virus integration site 1; WT, wild type.

Calcium handling is important in the contraction and diastole of muscle tissue.^31^ Many previous reports have shown that abnormal cardiac calcium handling is a key pathogenesis mechanism of HCM.^32^^,^^33^ To examine the calcium transients, we used nickase Cas9 to introduce calcium sensor green fluorescent calcium-modulated protein 6 fast type (GCaMP6f), which was inserted into the adeno-associated virus integration site 1 (AAVS1) locus of MYBPC3-KO and WT stem cell lines (Fig. 2A). At day 30, the MYBPC3-KO hiPSC-CMs showed increased Ca^2+^ transient amplitude compared with WT. On the contrary, the Ca^2+^ reuptake rate and release rate of MYBPC3-KO hiPSC-CMs increased at day 40, whereas the Ca^2+^ release amplitude decreased. Meanwhile, MYBPC3-KO hiPSC-CMs started to demonstrate prolonged decay times compared with WT hiPSC-CMs (Fig. 2B–D). These results suggest that MYBPC3-KO hiPSC-CMs show evident calcium transport problems. Increased contractility and diastolic insufficiency are the main features of HCM. To assess the impact of MYBPC3-KO on cardiac contractility, we evaluated the contractile force of hiPSC-CMs at day 20, 30, and 40. The contractility amplitude and myocardial diastolic time of MYBPC3-KO hiPSC-CM initially increased at day 30 compared with WT hiPSC-CMs, but at day 40, the contractility amplitude of MYBPC3-KO hiPSC-CM decreased, and myocardial diastolic time was markedly prolonged. The contractility amplitude showed no difference between MYBPC3-KO hiPSC-CMs and WT hiPSC-CMs at day 20 (Fig. 2E, F). These findings show that MYBPC3 deficiency would eventually result in an HCM phenotype in hiPSC-CMs, which is consistent with previous results of the typical phenotype caused by MYBPC3 disorders in patients. It can indicate the development of an HCM phenotype at day 30 and the development of heart failure at day 40.

### MYBPC3-KO hiPSC-CMs recapitulate the HCM phenotypes *in vitro*

**Figure 3:**
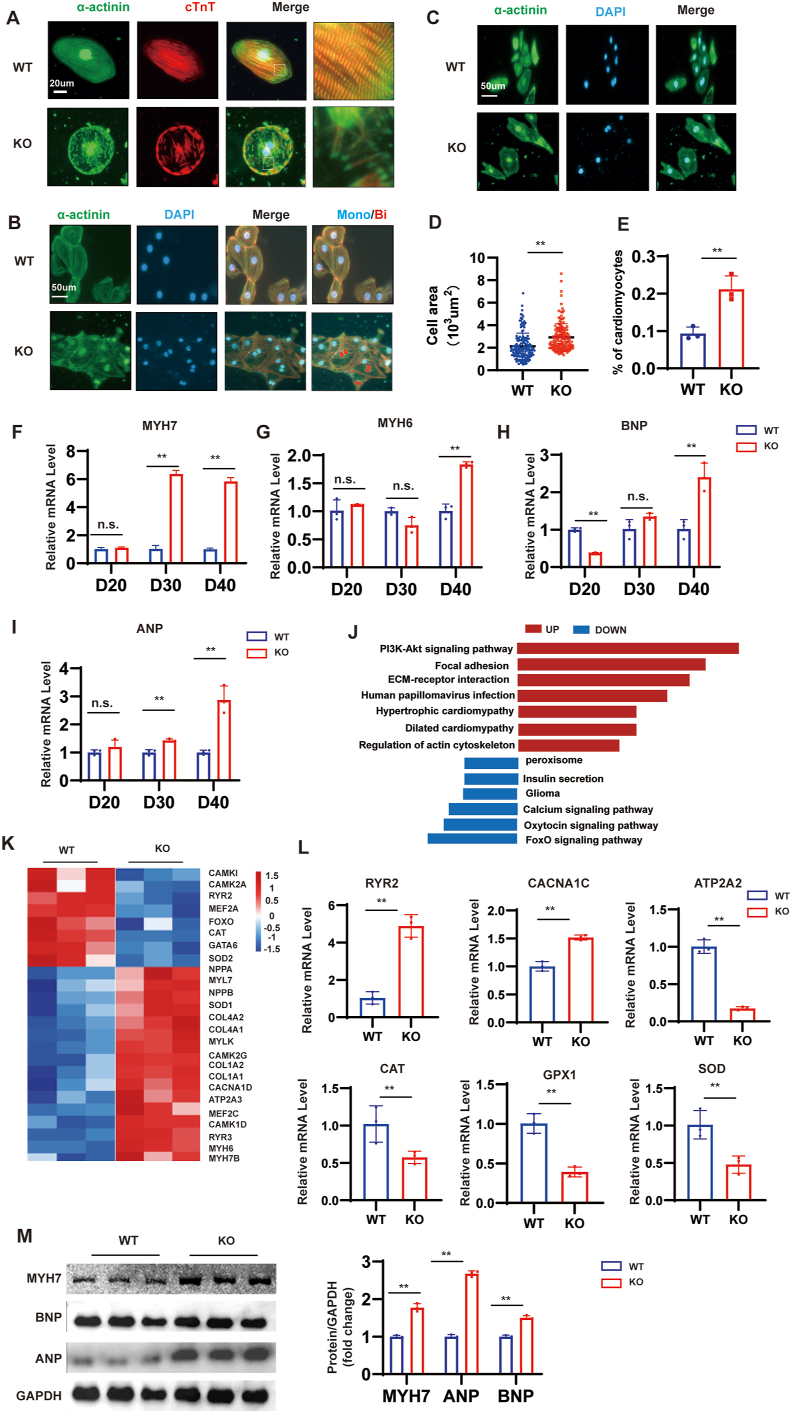
Phenotyping hypertrophic cardiomyopathy in hiPSC-CMs lacking MYBPC3. **(A)** The immunostaining for sarcomeric α-actinin (in green) and cTnT (in red) reveals disorganized sarcomeres in MYBPC3-KO hiPSC-CMs at day 40. Scale bar, 20 μm. **(B, D)** Visual representation of hiPSC-CMs stained with α-actinin, cTnT, and DAPI, along with the quantification of mono- and bi-nucleation in WT (total 540; *n* = 3180 per group) and MYBPC3-KO hiPSC-CMs (total 540; *n* = 3180 per group). Scale bar, 50 μm. **(C, E)** In contrast to WT hiPSC-CMs (*n* = 135), a notable majority of MYBPC3-KO hiPSC-CMs (*n* = 130) exhibited a heightened pattern in cell area sarcomeric α-actinin staining. Scale bar, 50 μm. **(F, G)** The quantitative real-time PCR focused on HCM-associated genes MYH7, MYH6, NPPB, and NPPA at intervals of 20, 30, and 40 days. **(J)** Utilizing Kyoto Encyclopedia of Genes and Genomes (KEGG) databases for enrichment analysis, it was discovered that routes associated with cardiomyopathy and oxidative stress became disturbed in MYBPC3-KO hiPSC-CMs. **(K)** Analysis of Gene Ontology (GO) enrichment revealed notable alterations in gene activity linked to cardiomyopathy and oxidative stress in MYBPC3-KO hiPSC-CMs. **(L)** The quantitative real-time PCR of genes RYR2, CACNA1C, ATP2A2, CAT, SOD, and GPX1 at day 40. **(M)** Western blotting of MYH7, ANP, and BNP levels in WT hiPSC-CMs versus MYBPC3-KO hiPSC-CMs at day 40 (*n* = 3). Data were shown as mean ± standard deviation. ∗*p* < 0.05; ∗∗*p* < 0.01; n.s. means no significance. hiPSC, human induced-pluripotent stem cell; CM, cardiomyocyte; MYBPC3, myosin binding protein C3; KO, knockout; cTnT, cardiac troponin T; HCM, hypertrophic cardiomyopathy; MYH6/7, myosin heavy chain 6/7; NPPA, natriuretic peptide A; NPPB, natriuretic peptide B; RYR2, ryanodine receptor 2; CACNA1C, calcium voltage-gated channel subunit alpha1 C; ATP2A2, ATPase sarcoplasmic/endoplasmic reticulum Ca^2+^ transporting 2; CAT, catalase; SOD, superoxide dismutase; GPX1, glutathione peroxidase 1.

During the development of HCM disease, CMs undergo a series of pathological changes, such as disorder in cardiomyocyte sarcomere arrangement, enlargement of cell area, and an increase in binuclear CMs.^34^^,^^35^ As previously reported, compared with WT hiPSC-CMs, the disarrayed sarcomeres of MYBPC3-KO hiPSC-CMs show a significant increase at day 30 post-differentiation (Fig. 3A). Strikingly, MYBPC3-KO hiPSC-CMs showed a 10% increase in bi-nucleation relative to WT hiPSC-CMs (Fig. 3B, D). Increased cardiomyocyte size is typical of HCM. MYBPC3-KO hiPSC-CMs increased by about 50 percent (Fig. 3C, E). These studies suggest that MYBPC3 deficiency presents pathological changes in the cell structure of HCM disease *in vitro*. Results revealed that myosin heavy chain 7 (MYH7), natriuretic peptide A (NPPA), and natriuretic peptide B (NPPB) were up-regulated in MYBPC3-KO hiPSC-CMs at day 30, and NPPA and NPPB significantly increased in MYBPC3-KO hiPSC-CMs at day 40, compared with the WT-hiPSC-CMs (Fig. 3F–I). To explore the effect of MYBPC3 deficiency upon HCM development at the cellular level, we performed a global transcriptome analysis comparing MYBPC3-KO hiPSC-CMs and WT-hiPSC-CMs to establish these pathways at day 40. We can see that hypertrophy-related genes and pathways were elevated (Fig. 3J, K). We also tested calcium channels and oxidative stress-related genes. Results showed that ryanodine receptor 2 (RYR2) and calcium voltage-gated channel subunit alpha1 C (CACNA1C) expression were up-regulated in the KO group, while ATPase sarcoplasmic/endoplasmic reticulum Ca^2+^ transporting 2 (ATP2A2), catalase (CAT), superoxide dismutase (SOD), and glutathione peroxidase 1 (GPX1) expression were down-regulated (Fig. 3L). We then tested the protein-related levels of gene expression at day 40. We found that the expression of MYH7, ANP, and BNP protein was increased in the MYBPC3-KO hiPSC-CM compared with WT hiPSC-CMs (Fig. 3M). Taken together, these results show that the levels of genes and proteins associated with hypertrophy were consistent with previous studies.^36^

### MYBPC3-deficient CMs activate oxidative stress

**Figure 4:**
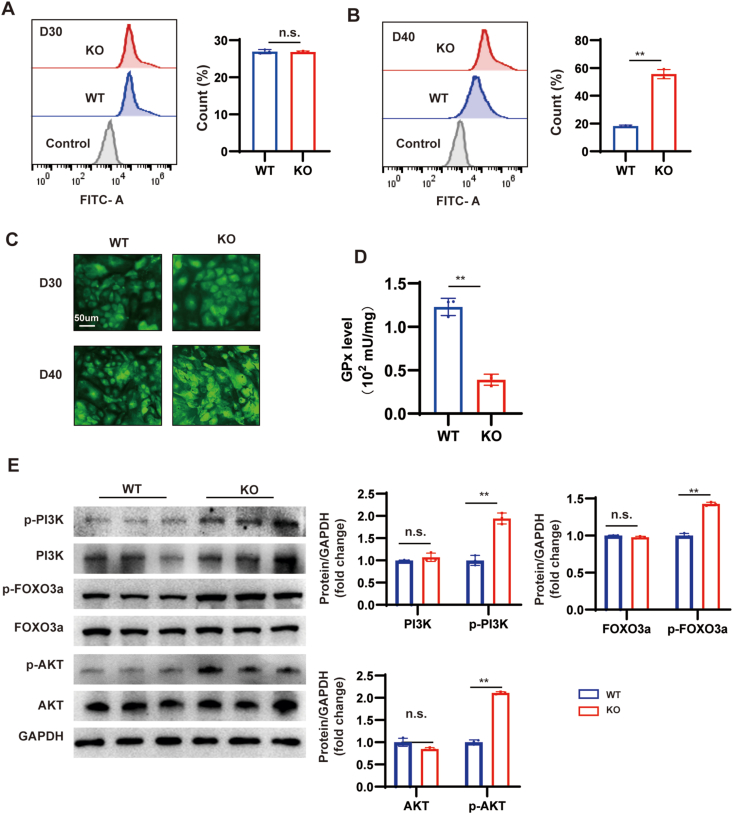
MYBPC3 deficiency causes excessive oxidative stress. **(A, B)** The green intensity of cell ROS was measured by flow cytometry in MYBPC3-KO hiPSC-CMs at day 30 and 40, alongside WT hiPSC-CMs (*n* = 3; 10,000 cells per sample). **(C)** A comparison was made between the green intensity of ROS in MYBPC3-KO hhiPSC-CMs and WT hiPSC-CMs at day 30 and 40. Scale bar, 50 μm. **(D)** GPx level was significantly decreased at day 40 in MYBPC3-KO and WT hiPSC-CMs. **(E)** Western blotting and quantification of oxidative stress pathway signaling (PI3K, p-PI3K, AKT, p-AKT, FOXO3a, p-FOXO3a) in WT and MYBPC3-KO hiPSC-CMs at day 40. ∗*p* < 0.05; ∗∗*p* < 0.01; n.s. means no significance. hiPSC, human induced-pluripotent stem cell; CM, cardiomyocyte; MYBPC3, myosin binding protein C3; KO, knockout; GPx, glutathione peroxidase; PI3K, phosphatidylinositol-3-kinase; AKT, protein kinase B; FOXO3a, forkhead box O 3a; ROS, reactive oxygen species.

Oxidative stress is thought to be a key causative factor in myocardial hypertrophy. In the RNA sequencing results, it was found that the oxidative stress pathway was activated. Because there were no abnormalities in related indicators such as calcium flux and contractility at day 20, we tested the changes in indicators at day 30 and day 40 to explore the mechanism of disease development. Alterations in cellular ROS levels were initially evaluated using flow cytometry. At day 30, the ROS levels of MYBPC3-KO hiPSC-CMs remained unchanged, in contrast to a notable rise at day 40 (Fig. 4A, B). The green fluorescent probe assay showed a positive intensity of cell ROS in the MYBPC3-KO hiPSC-CMs at day 40 and no change at day 30 (Fig. 4C). Glutathione peroxidase (GPX) can scavenge free radicals and maintain redox balance. However, the amount of GPX decreased at day 40 (Fig. 4D). ROS-sensitive signaling pathways, such as phosphatidylinositol-3-kinase (PI3K)/protein kinase B (AKT), are activated by stimuli, such as pressure overload, mechanical stretches, and G protein-coupled receptor (GPCR) agonists, playing a significant role in the development of cardiac hypertrophy.^37^ RNA sequencing showed that the PI3K/AKT signaling pathway was up-regulated, while the forkhead box O (FOXO) signaling pathway was down-regulated. The results showed that the PI3K/AKT signaling pathway was activated, and forkhead box O3a (FOXO3a) was decreased (Fig. 4E).

### MYBPC3-deficient CMs develop mitochondrial dysfunction

**Figure 5:**
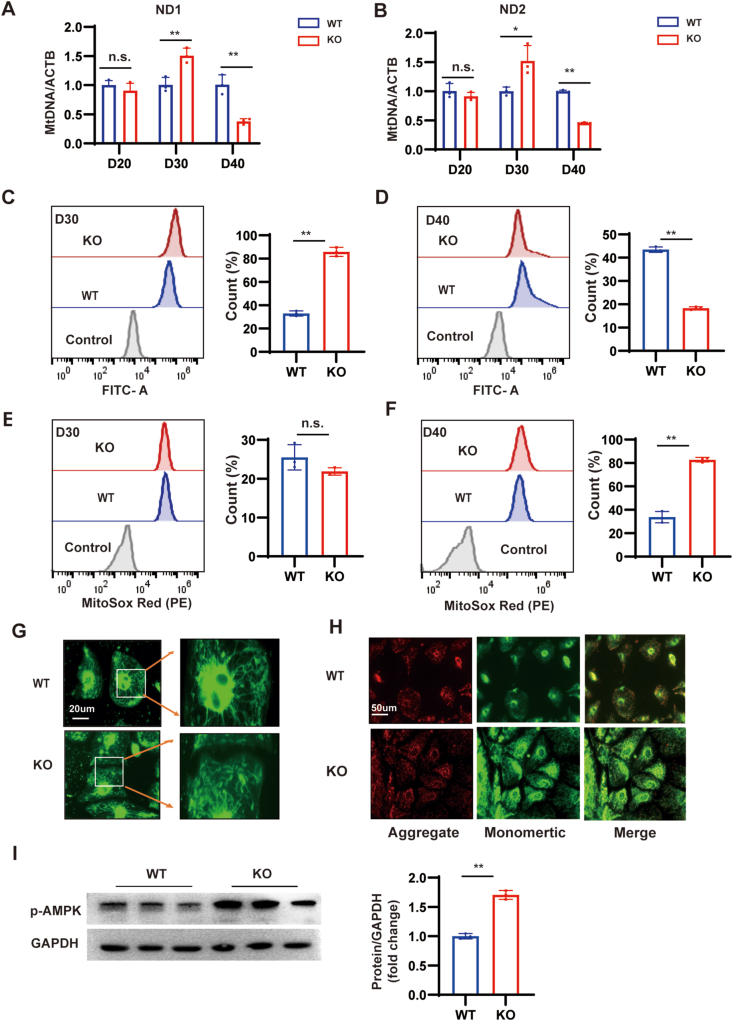
MYBPC3 deficiency causes mitochondrial dysfunction. **(A, B)** Quantitative PCR analysis of ND1 and ND2 to nuclear DNA (β-actin) ratio at day 20, 30, and 40 (*n* = 3). **(C–F)** Mitotracker Green and MitoSOX Red were measured via flow cytometry in MYBPC3-KO hiPSC-CMs on day 30 and 40, in comparison to WT hiPSC-CMs (*n* = 3). **(G)** Representative immunofluorescence staining images of Mitotracker. Scale bar, 20 μm. **(H)** Immunofluorescence analysis of JC-1 showed a rise in mitochondrial monomers (indicated by green fluorescence) and a steady reduction in mitochondrial aggregates (shown as red fluorescence) in MYBPC3-KO hiPSC-CMs relative to WT on the 40th day. Scale bar, 50 μm. **(I)** A typical western blotting analysis of p-AMPK in MYBPC3-KO hiPSC-CMs at day 40 was performed, in comparison to WT hiPSC-CMs. ∗*p* < 0.05; ∗∗*p* < 0.01; n.s. means no significance. hiPSC, human induced-pluripotent stem cell; CM, cardiomyocyte; MYBPC3, myosin binding protein C3; KO, knockout; ND1, NADH dehydrogenase subunit 1; ND2, NADH dehydrogenase subunit 2; AMPK, AMP-activated protein kinase.

Mitochondria are important in maintaining cellular energy metabolism, and excessive Ca^2+^ can lead to mitochondrial damage, especially ROS. To further explore the changes in mitochondria, we examined NADH dehydrogenase subunit 1 (ND1) and NADH dehydrogenase subunit 2 (ND2) at day 20, 30, and 40, and found that there was no change at day 20, and at day 30, ND1 and ND2 increased in MYBPC3-KO hiPSC-CMs; and conversely, they decreased at day 40 (Fig. 5A, B). To further understand the alterations in mitochondria, we measured the mitochondrial content and mitochondrial-specific ROS at day 30 and day 40. At day 30, mitochondrial content increased compared with WT, while MitoSOX remained at the same level. Mitotracker levels decreased on day 40, whereas MitoSOX levels noticeably increased (Fig. 5C–F). These findings revealed that following MYBPC3-KO, at day 30, mitochondrial function was active, but at day 40, mitochondrial function was significantly impaired. We next assessed mitochondrial morphology using Mitotracker at day 40. In contrast to the typical linear arrangement of mitochondria along the sarcomere in WT hiPSC-CMs, mitochondria in MYBPC3-KO hiPSC-CMs showed a uniquely fragmented and punctate pattern and were irregularly distributed throughout the cytoplasm (Fig. 5G). Mitochondrial membrane potential (Δψm) is an important indicator for detecting mitochondrial function. To investigate the impact of MYBPC3 deficiency on mitochondrial function, JC-1 is an ideal fluorescent probe for the detection of mitochondrial membrane potential. Notably, on day 40, the ratio of aggregate to monomeric JC-1 fluorescence in the MYBPC3-KO hiPSC-CMs significantly reduced compared with that of the WT hiPSC-CMs (Fig. 5H). Mitochondrial damage and decreased numbers can lead to insufficient energy production by CMs. Phospho-AMP-activated protein kinase (p-AMPK) protein level was significantly higher in MYBPC3-KO hiPSC-CMs at day 40 (Fig. 5I).

### Melatonin alleviates HCM through PI3K/AKT/FOXO3a signaling pathways

**Figure 6:**
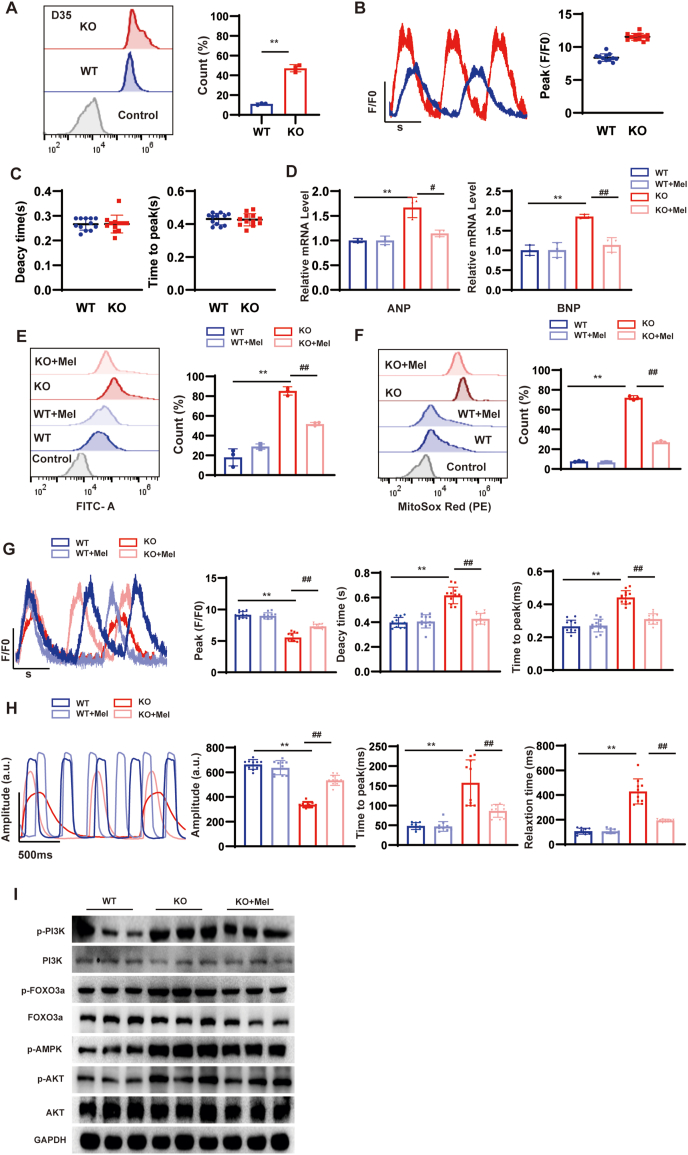
Melatonin alleviates hypertrophic cardiomyopathy. **(A)** The green intensity of cell ROS was measured by flow cytometry in MYBPC3-KO hiPSC-CMs at day 35, in comparison to WT hiPSC-CMs (*n* = 3; 10,000 cells per sample). **(B, C)** Quantification of peak, time to peak, and calcium transient duration in WT and MYBPC3 KO hiPSC-CMs at day 35 (*n* = 12 cells per group). **(D)** Quantitative real-time PCR of NPPA and NPPB in WT, MYBPC3-KO, and MYBPC3-KO + melatonin hiPSC-CMs at day 40. **(E, F)** Cell ROS Green and MitoSOX Red were measured using flow cytometry to assess fluorescence intensity in WT, MYBPC3-KO, and MYBPC3-KO + melatonin hiPSC-CMs at day 40. **(G)** The peak, time to peak, and calcium decay time were measured in WT-GCaMP, MYBPC3-KO-GCaMP, WT + melatonin, and MYBPC3-KO-GCaMP + melatonin hiPSC-CMs at day 40 (*n* = 12 cells per group). **(H)** Quantification of amplitude, time to peak, and relaxation time at day 40 (*n* = 12 cells per group). **(I)** Representative Western blot of oxidative stress pathway signaling at day 40. ∗*p* < 0.05; ∗∗*p* < 0.01; n.s. means no significance. ∗∗*p* versus the corresponding WT group; ^#^*p* versus the KO group. MYBPC3, myosin binding protein C3; KO, knockout; hiPSC, human induced-pluripotent stem cell; CM, cardiomyocyte; NPPA, natriuretic peptide A; NPPB, natriuretic peptide B; GCaMP, green fluorescent calcium-modulated protein; ROS, reactive oxygen species.

To verify the relationship between ROS and HCM, we measured ROS and calcium flux at day 35, and the results showed that ROS levels were elevated in KO at day 35 (Fig. 6A), but the Ca^2+^ transient was consistent with the levels at day 30 (Fig. 6B, C). This suggests that ROS preceded symptoms of heart failure. Melatonin is a ubiquitous multifunctional molecule with most of the desirable properties of a good antioxidant. To date, the amount of data on the protective effect of melatonin against oxidative stress has been overwhelming.^38^ In this experiment, we added melatonin (10 μmol) for 5 days starting at day 30 to further explore its role in the development of HCM. After the addition of melatonin, we could find that the relative expression of fetal genes NPPA and NPPB was significantly reduced in MYBPC3-KO hiPSC-CMs relative to WT at day 40 (Fig. 6D). Flow cytometry results showed a significant improvement in cellular ROS and MitoSOX levels (Fig. 6E, F). After treating with melatonin for 5 days, the Ca^2+^ transient was considerably decreased, and the maximal Ca^2+^ amplitude was increased in MYBPC3-KO hiPSC-CMs at day 40 (Fig. 6G). After melatonin therapy, the contractility amplitude of MYBPC3-KO hiPSC-CMs was restored to approximately 72% compared with WT (Fig. 6H). Western blotting results following melatonin therapy demonstrated that melatonin administration decreased PI3K phosphorylation, AKT phosphorylation, and FOXO3a phosphorylation (Fig. 6I). The increased non-phosphorylated FOXO exerted an antioxidant effect.

## Discussion

The MYBPC3 mutation is widely recognized as the predominant genetic factor in HCM. As mentioned above, MYBPC3-deficient CMs showed an enlargement in cell surface area, accompanied by an increase in the quantity of genes and proteins associated with hypertrophy. In the MYBPC3-KO model, a higher amplitude of calcium transients was subsequently observed in MYBPC3-deficient hiPSC-CMs at day 30 of cardiac differentiation compared with WT hiPSC-CMs. Similarly, there was a notable rise in the amplitude of contractility in MYBPC3-deficient hiPSC-CMs. At day 40, MYBPC3-deficient hiPSC-CMs displayed decompensated state heart failure, marking the end-stage of the disease. Additionally, it was observed that ROS levels stabilized in the compensatory phase, and by the end of HCM, ROS became excessive, leading to mitochondrial damage and unbalanced energy metabolism. When melatonin was added for treatment, it was found that the intracellular level of oxidative stress was considerably decreased, and the maximal amplitude of calcium handling was increased. After melatonin therapy, the contractility amplitude of MYBPC3-KO hiPSC-CMs recovered to roughly 72% of the WT level.

A multitude of research indicates that oxidative stress can be balanced in favor of slowing the progression of cardiac hypertrophy.^39^ Mitochondria are sites in CMs that produce ROS. At day 30, to effectively enhance myocardial contractility, CMs need more mitochondria to meet high energy demands.^40^ At day 35, the MYBPC3-KO hiPSC-CMs showed increased Ca^2+^ transient amplitude compared with WT, and ROS were significantly increased compared with the WT group. In the end stage of HCM, the calcium release time and decay time were significantly prolonged, and the contractility amplitude of MYBPC3-KO hiPSC-CMs decreased. In the end stage of HCM, the calcium release time and decay time were significantly prolonged, and the contractility amplitude of MYBPC3-KO hiPSC-CMs decreased. Calcium recovery was reduced, and there was cytoplasmic calcium overload. Cellular calcium overload leads to an increase in mitochondrial calcium, leading to mitochondrial dysfunction. Increased cytoplasmic calcium overload in CMs can subsequently lead to mitochondrial calcium overload, dysfunction, and oxidative stress. As cardiac contraction increases, intracytoplasmic Ca^2+^ levels increase, allowing more Ca^2+^ to enter the mitochondria and increasing the activity of ATP synthase and electron transport chain accelerating ATP production. HCM has a persistent increase in contractility, with persistent Ca^2+^ entering the mitochondria, leading to mitochondrial calcium overload, increased ROS production, and oxidative stress.^41^^,^^42^ Thus, during the compensatory phase of HCM, persistently high contractility leads to an increase in intracytoplasmic calcium levels, which in turn leads to an increase in the level of oxidative stress induced by mitochondrial calcium overload. The accumulation of ROS can result in the activation of the mitochondrial membrane permeability transition pore (MPT), which in turn causes a reduction in mitochondrial transmembrane potential (△ψm).^43^ We found that the mitochondrial fluorescence intensity and mtDNA/nDNA ratio of MYBPC3-KO hiPSC-CMs significantly reduced at day 40 compared with WT cells, further indicating mitochondrial damage in CMs after MYBPC3 knockout. Additionally, we found that the mitochondrial morphology of KO cells was abnormal at day 40. The mitochondria of normal WT cells were more linear, while those in KO cells were more punctate and fragmented. Previous studies have shown that punctate or fragmented mitochondria are more common in patients with heart failure. In addition, CaMKII activated by ROS may boost Ca^2+^ overload by raising late Na ^+^ current, leading to an increase in intercellular Na^+^ and Ca^2+^ entry by the Na^+^/Ca^2+^ exchanger (NCX).^44^ Alteration of sarcoplasmic reticulum Ca^2+^-ATPase (SERCA) activity by the redox system may also lead to abnormal calcium handling.^45^ This creates a vicious circle in which intracytoplasmic calcium overload causes mitochondrial damage and then results in elevated ROS levels, which in turn affect calcium handling and myocardial contractility, leading to the so-called ROS-induced ROS release in mitochondria. The adequate energy supply of CMs is the most important basis for maintaining the heart's pumping function, and mitochondria are the vital sites for energy production. As an energy sensor, AMPK is critical in cellular energy metabolism. The expression of p-AMPK was significantly increased in MYBPC3-KO CMs during the test. When the energy supply to KO cells is reduced, energy-consuming physiological activities such as SERCA2a, NCX, and ATP2A4 are all inhibited to some extent. This leads to a further increase in intracellular calcium concentrations, which can result in a more severe intracellular calcium overload. Increased calcium ions then enter the mitochondria, causing further damage, which, in turn, reduces the energy supply. Cardiac hypertrophy is an adaptive process. Thus, in the early stages of HCM, the formation of a small amount of oxygen radicals may not be sufficient to cause the occurrence of oxidative stress. However, they may produce redox-sensitive signals that activate the hypertrophic process of the myocardium. On the other hand, high levels of oxygen radicals due to prolonged pathological stimulation can lead to oxidative stress, poor cardiac remodeling, cardiac dysfunction, and heart failure.^46^

Melatonin receptor MT1 (melatonin receptor type 1) and MT2 (melatonin receptor type 2) belong to the 7-transmembrane class A GPCR superfamily, which is widely distributed in the brain, heart, liver, spleen, kidney, *etc.*, and its endogenous ligand is melatonin, which is closely related to various life activities, such as reproduction, neuroregulation, immune regulation, cardiovascular function.^47^ Melatonin can increase the antioxidant enzymes in cells, including superoxide dismutase, glutathione peroxidase, and glutamate cysteine synthase; melatonin can directly bind to reactive oxygen species and reactive nitrogen free radicals; and melatonin quickly crosses the mitochondrial membrane and accumulates in the mitochondria to bind in concentrations up to hundreds of times higher than conventional antioxidants.^48^^,^^49^ Melatonin is an endogenous hormone produced by the pineal gland and released only at night.^50^ Epidemiological studies have shown reduced levels of circulating melatonin in patients with heart failure.^51^ Meanwhile, in patients with heart failure, melatonin levels are also related to remodeling after cardiac resynchronization therapy.^52^ Many studies have proven that melatonin is important in relieving pathological myocardial hypertrophy.^37^ Sensitive ROS signaling pathways, such as PI3K/AKT, activated by stress overload, such as tensile mechanical stimulation, play an important role in the development of myocardial hypertrophy.^53^ FOXO regulates key physiological functions, including stress response, cell cycle progression, protein degradation, and apoptosis.^54^ There are four members of the FOXO family in mammals, namely, FOXO1, FOXO3a, FOXO4, and FOXO6; FXOXO1 is highly expressed in adipose tissue, heart, brain, and skin, FOXO3a is highly expressed in skeletal muscle, myocardium, and neurons, FOXO4 is highly expressed in myocardium, and FOXO6 is only expressed in specific regions of the brain; FOXO3a is the most abundantly expressed subtype in the heart and is the most important potential target for the treatment of cardiac diseases.^53^^,^^55^ RNA sequencing showed that PI3K/AKT/FOXO3a signaling pathway was activated. When PI3K/AKT is activated, phosphorylated FOXO3a increases, and its antioxidant effect is weakened; FOXO3a promotes the expression of antioxidant genes and decreases ROS levels, which further inhibit cardiomyocyte hypertrophy.^56^ Previous studies have shown that melatonin can modulate PI3K/AKT/FOXO3a signaling pathways to exert its effects. In this experiment, after melatonin treatment, we could find that melatonin inhibited PI3K/AKT/FOXO3a signaling, and flow cytometry showed a decrease in cellular ROS and MitoSOX levels and an improvement in calcium flux and contractility. We can conclude that melatonin can improve the development of HCM disease by inhibiting the PI3K/AKT/FOXO3a signaling pathways.

In conclusion, the early diagnosis of oxidative stress-related diseases such as heart disease remains the primary challenge for society. HCM is a complex process that ultimately leads to heart failure. Studies have shown that unregulated ROS are involved in inducing many diseases. In the development of HCM from compensated to decompensated heart failure, we find that ROS levels increase, mitochondrial damage occurs, and the classical pro-oxidative pathway PI3K/AKT/FOXO3a is activated. The antioxidant melatonin can balance oxidative stress by inhibiting the PI3K/AKT/FOXO3a pathways. Moreover, a detailed understanding of ROS in HCM has great potential therapeutic value for cardiac diseases.

## CRediT authorship contribution statement

**Jinhua Cao:** Formal analysis, Project administration, Investigation, Writing – original draft. **Yafei Zhai:** Conceptualization, Writing – original draft, Methodology, Supervision, Software. **Ke Li:** Conceptualization, Software, Methodology. **Jiajv Li:** Methodology, Investigation. **Xiaoxu Tian:** Data curation, Software. **Jianchao Zhang:** Methodology, Data curation. **Shuang Li:** Methodology, Formal analysis. **Mengduan Liu:** Investigation, Formal analysis. **Xiaowei Li:** Resources, Software. **Jianzeng Dong:** Writing – review & editing, Project administration, Resources. **Xiaofang Wang:** Funding acquisition, Writing – review & editing, Project administration.

## Funding

This work was financially supported by the 10.13039/501100001809National Natural Science Foundation of China (No. 82070824) and the Henan Province Young and Middle-aged Health Science and Technology Innovation Leading Talent Training Project (China) (No. YXKC2022017).
