# Supplementary material for: ROS accelerates the progression of hypertrophic cardiomyopathy
Source: Genes Dis. 2025 Jun 27;13(1):101741. doi: 10.1016/j.gendis.2025.101741 (PMC12555768; doi:10.1016/j.gendis.2025.101741)
Supplement: Multimedia component 1 [file mmc1.docx]

**Table S1**

**Primer sequences used for q-PCR**

| Gene | Forward | Reverse |
| --- | --- | --- |
| GAPDH | GGAGCGAGATCCCTCCAAAAT | GGCTGTTGTCATACTTCTCATGG |
| MYH7 | GGCAAGACAGTGACCGTGAAG | CGTAGCGATCCTTGAGGTTGTA |
| MYH6 | TCTCCGACAACGCCTATCAGTAC | GTCACCTATGGCTGCAATGCT |
| ANP | ACAATGCCGTGTCCAACGCAGA | CTTCATTCGGCTCACTGAGCAC |
| BNP | TCTGGCTGCTTTGGGAGGAAG | CCTTGTGGAATCAGAAGCAGGTG |
| ND1 | ATGGCCAACCTCCTACTCCTCATT | TTATGGCGTCAGCGAAGGGTTGTA |
| ND2 | CCATCTTTGCAGGCACACTCATCA | ATTATGGATGCGGTTGCTTGCGTG |
| ACTB | CATGTACGTTGCTATCCAGGC | CTCCTTAATGTCACGCACGAT |
| ATP2A2 | GATCACACCGCTGAATCTG | AGTATTGCGGGTTGTTCCAG |
| RYR2 | AGAACTTACACACGCGACCTG | CATCTCTAACCGGACCATACTGC |
| CACNA1C | CAGAGGCTACGATTTGAGGA | GCTTCACAAAGAGGTCGTGT |
| SOD | CTGGACAAACCTCAGCCCTAAC | AACCTGAGCCTTGGACACCAAC |
| CAT | GTGCGGAGATTCAACACTGCCA | CGGCAATGTTCTCACACAGACG |
| GPX1 | GTGCTCGGCTTCCCGTGCAAC | CTCGAAGAGCATGAAGTTGGGC |

**Antibody**

| Antibody | Product Information | |
| --- | --- | --- |
| GAPDH | 60004-1-Ig | Proteintech |
| MYL2 | 10906-1-Ig | Proteintech |
| ACTININ | Ab137346 | Abcam |
| cTNT | Sc515859 | Santacruz |
| MYH7 | Sc71575 | Santacruz |
| NT-proBNP | Ab13115 | Abcam |
| p-CAKMII | #12716 | Cell signaling |
| PI3K | #4249s | Cell signaling |
| p-PI3K | #17366 | Cell signaling |
| AKT | 10176-2-Ig | Proteintech |
| p-AKT | 66444-1-Ig | Proteintech |
| FOXO3a | 66428-1-Ig | Proteintech |
| p-FOXO3a | 28755-1-Ig | Proteintech |
| p-AMPK | #50081s | Cell signaling |
